# Chewing and Attention: A Positive Effect on Sustained Attention

**DOI:** 10.1155/2015/367026

**Published:** 2015-05-17

**Authors:** Yoshiyuki Hirano, Minoru Onozuka

**Affiliations:** ^1^Research Center for Child Mental Development, Graduate School of Medicine, Chiba University, 1-8-1 Inohana, Chuo-ku, Chiba 260-8670, Japan; ^2^Research Program for Carbon Ion Therapy and Diagnostic Imaging Research, Center for Charged Particle Therapy, National Institute of Radiological Sciences, 4-9-1 Anagawa, Inage-ku, Chiba 263-8555, Japan; ^3^Department of Judo Therapy and Medical Science, Nippon Sport Science University, 7-1-1 Fukuzawa, Setagaya-ku, Tokyo 158-8508, Japan

## Abstract

Chewing is crushing food not only to aid swallowing and digestion, but also to help stress relief and regulate cognitive function, especially in attention. It is well known that chewing gum is used for sleepiness prevention during work, learning, and driving, suggesting a link between chewing and sustained attention. We hypothesized that chewing elevates attention and/or alertness, leading to improvements in cognitive performance. We carried out a systematic review of the PubMed database. We inspected the attributes of effects on attention in studies investigating the effects of chewing on attention or alertness conducted with pre-post design in healthy subjects, except elderly. We identified 151 references, 22 of which were included: 14 (64%) showed positive attributes of effects on attention, 1 (5%) showed negative attributes of effects on attention, 5 (23%) showed both positive and negative attributes of effects on attention, and 2 (9%) showed no significant attributes of effects on attention. Thus, positive attributes of effects of chewing on attention, especially on sustained attention, were shown in over half of the reports. These effects also appeared with improvement in mood and stress relief and were influenced by time-on-task effect. Further studies are needed, but chewing could be useful for modifying cognitive function.

## 1. Introduction

The most dominant function of chewing (or mastication) is thought to be crushing of food to aid swallowing and digestion. Besides this essential role, recent studies have mentioned a unique function that affects brain function. For example, bruxism, which is involuntary grinding of teeth typically during sleep, is thought to be caused by psychological stress [1, 2]. Furthermore, chewing gum is used for maintaining alertness and avoiding sleepiness while operating a vehicle or studying. Also, adverse oral health such as periodontitis [3] and tooth loss [4] may be a risk factor of cognitive decline in elderly. These findings support the concept that chewing is strongly associated with cognitive function such as learning and memory and keeping and increasing attention [5].

This relationship between chewing and cognitive functions was empirically estimated a long time ago. In 1939, Hollingworth [6] reported that chewing increased relaxation and the performance of cognitive function like number-checking and typing. This relaxation effect was investigated by means of electroencephalography (EEG), and regular gum chewing was shown to increase alpha power after chewing compared with control at almost all positions [7]. After that, additional EEG experiments were conducted under conditions of chewing gum with flavor or almost no taste and odor [8, 9]. The results showed that alpha and beta activity patterns were not consistent, although chewing flavored gum consistently increased beta activity and induced arousal effects. Thereafter, more than twenty studies investigated the effects of chewing on cognitive function using modern neuropsychological testing, the first one being carried out by Wilkinson et al. in 2002 [10]. They reported improvement of performance of episodic memory and spatial and numeric working memory in the chewing group. However, no improvement was observed in attention. After that, Tucha et al. [11] presented controversial results in their replication study. They reported that chewing increased sustained attention, although no beneficial effect was found on memory, and there were aversive effects on alertness and flexibility. Since those studies, the issue concerning the existence and attributes of effects of chewing has remained a subject of discussion up to the present. Later on, several studies investigated the relationship between chewing and cognitive functions using functional magnetic resonance imaging (fMRI) and EEG [12–15] to elucidate the mechanisms of these effects. However, the reported effects of chewing on cognitive functions have been rather inconsistent and have still not been fully clarified. With regard to the results, we hypothesized that chewing affects attention and/or alertness, consequently leading to improvements of cognitive performance. Therefore, we reviewed the literature in respect to the effects of chewing on aspects of attention, and we discussed the existence and attributes of these effects.

## 2. Methods

### 2.1. Search Strategy

A systematic search strategy was used to identify appropriate publications. We conducted online search at the PubMed online database using the terms (chewing OR mastication) AND (attention OR alertness OR cognitive) NOT (“oral health” OR review OR elderly OR disorders) on August 5th, 2014, with no time span specified for publication date. A total of 151 hits came back.

### 2.2. Selection Criteria

Studies were included if they met the following criteria: (i) reported in an original paper, (ii) examined the effect of chewing in healthy children or adults, but not in the elderly, to rule out the possible influence of oral health and cognitive impairments, (iii) evaluated the efficacy of attention, including alertness, vigilance, executive control, and reaction time, compared with no gum chewing conditions, and (iv) written in English. After applying these selection criteria, 22 papers were included in the current review.

### 2.3. Variables of Interest

The following variables were examined for each article included in the review: (i) cognitive tests and psychological rating scales, if applicable, (ii) chewing objects, (iii) summary of results compared with nonchewing condition unless stated, and (iv) attributes of effects on attention.

## 3. Results and Discussion

The 22 articles were checked for cognitive tests and psychological rating scales, chewing objects, summary of results, and attributes of effects on attention (see Table 1). First, we focused on the most interesting variable, the attributes of effects on attention, categorizing them by the direction of effects. As shown in Table 1, more than half of the reports indicated positive attributes of effects on attention, with 14 (64%) showing positive (at least somewhat), 1 (5%) showing negative, 5 (23%) showing both positive and negative attributes of effects on attention, and 2 (9%) showing no significance. The appearance of reports presenting negative attributes showed a decreasing trend with recent experimental conditions. Next, we discussed the factors of mood and time-on-task effects on the results according to the summaries of results in order to understand the mechanisms.

With regard to mood, Smith [16] examined memory, intelligence test, alertness, and mood in a single study. He reported that alertness and hedonic tone in addition to intelligence test were improved, although memory showed no improvement. The following study by Smith [17] added improved selective and sustained attentions while the hedonic tone benefit had disappeared. Also, increased pretest alertness and hedonic tone and reduced posttest anxiety in mood were indicated, but without benefit for attention in another report by Smith [18]. He mentioned that two types of mechanisms were activated by chewing [16]. One was related to the mobilization of energetic resources, in particular facial muscles [19], and another was related to neurotransmitter function, specifically the 5-HT descending inhibitory pathway [20]. Scholey et al. [21] reported that chewing induced better performance on the test battery requiring memory and attention, with increased alertness and reduced state anxiety, stress, and saliva cortisol. They speculated that the mechanism of cognitive performance enhancement was secondarily induced by relief of mental stress [22], in addition to increased heart rate [10], cerebral blood flow [23], and brain activity [24] due to chewing. Thereafter, antistress effects were not replicated, but increased alertness was observed following the cognitive stressor test [25] and acute social stress test [26], which was suspected to be induced by greater cerebral activity following the chewing of gum [27]. Sakamoto et al. [14, 15] have reported the influences of chewing on the central nervous system by measuring reaction time (RT) and event-related potentials (ERPs). They suggested that chewing affects the state of arousal via the ascending reticular activating system, and this accelerates cognitive processing. More recently, Johnson et al. [28] found improvement in sustained attention covaried with subjective alertness, strongly supporting the hypothesis that chewing elevates attention and/or alertness, consequently leading to improvements in cognitive performance.

Another factor, the time-on-task effect in relation to the effect of chewing on attention, was discussed. Tucha and Simpson [29] proposed that time is an important factor in the psychodynamics of gum chewing. They put forward this new idea that it is one of the reasons for the difficulties in replicating the results of studies, in addition to the brand of gum, familiarity with gum, the experimental design, and statistical analysis. Tänzer et al. [30] examined the concentration performance in 8-9-year-old children using a 16-minute test. For the first 12 minutes, classes who did not chew gum performed better but were then overtaken by classes chewing gum, showing the interaction between chewing condition and time. After that, Allen and Smith [31] examined the time-on-task effect within each individual performance task, confirming this effect on vigilance reaction time. More recently, Morgan et al. [32] also reported that correct reaction times were significantly shorter in the latter stages of the task, in addition to the decline in both performance and subjective alertness in the chewing gum group. Johnson et al. [28] also reported the existence of time-on-task effect and initial impairment of attention. Tucha and Simpson [29] speculated that participants might be distracted by dual task interference [33] induced by gum chewing during early stages of cognitive tasks or that certain biological processes (e.g., increase of regional cerebral blood flow) have to add up or reach a certain threshold to facilitate cognitive processing. In this view, as a result of the study, the better performance of working memory task in chewing condition at the last stage could be explained [13].

The mechanisms of the beneficial effects of chewing on attention have been discussed for a long time, and they have been estimated as being derived from increases of cerebral blood flow and brain activity [12, 24, 27], cerebral blood flow [23, 34], cardiovascular system [10, 17, 35, 36], ascending reticular activating system [14, 15], glucose delivery [37], and flavors [11]. Recently, Hasegawa et al. [34] assumed that taste and odor can influence brain activation during chewing in sensory, cognitive, and motivational processes rather than in motor control, although some studies confirmed the beneficial effects on attention with tasteless and odorless gum base [12, 14, 15]. Allen and Smith [38] reported that a benefit for alertness was shown in persons with positive and neutral demand characteristics, but a positive effect on response organization was observed with demand characteristics [39] and the pretest attitude to gum. More recently, Yu et al. [40] demonstrated an fMRI study showing that gum chewing inhibited functional connectivity between the left anterior insular and the dorsal anterior cingulate cortex and functional connectivity from the superior temporal sulcus to the left anterior insula when activated by noise. They stated that gum chewing relieves stress by attenuating the sensory processing of external stressor and by inhibiting the propagation of stress-related information in the brain stress network. Allen et al. [35] reported that chewing can alter central and sympathetic nervous system activity associated with vigilance performance. The transient effect in their study was consistent with the short-lived effect of chewing gum on hits in the vigilance task.

In conclusion, many of the studies indicated that chewing exerts a positive effect on attention, and especially on sustained attention, in addition to improved mood and stress relief. Also, the effect seems to be influenced by time-on-task effect and do not last so long, such as 15–20 [41] or more than 30 minutes [29] after chewing, and then the mechanisms of the effects were not yet fully elucidated. Further studies are needed, but chewing could be useful as an easy method for modifying cognitive function on a daily basis and not be demanding physically and mentally.

## Figures and Tables

**Table 1:** Studies on the effects of chewing on attention.

Study	Cognitive tests and psychological rating scales	Chewing objects	Summary of results (compared with nonchewing condition unless stated)	Attributes of effects on attention
Wilkinson et al. (2002) [10]	15 tests on memory and attention	Sugar-free spearmint gum	Improvements of scores on episode and working memory and simple reaction time. Elevation of heart rate. No significant differences in attention.	Not significant

Tucha et al. (2004) [11]	12 tests on memory and attention	Sugar-free spearmint gum and sugar-free tasteless gum	Shortening of reaction time on sustained attention, prolongation of reaction time on alertness, and increase of number of errors on flexibility. No significant differences in memory and pulse rate.	Positive or negative depending on task

Stephens and Tunney (2004) [37]	8 tests on memory and attention	Sugar-free mint flavored gum	Improvements of scores on episode and working memory, attention, and processing speed. No significant differences in executive function.	Positive

Kohler et al. (2006) [36]	Psychomotor vigilance, tracking, and grammatical reasoning and alertness	Parafilm (sugar-free, tasteless)	Not significant or detrition of performance of speed and accuracy on simple and complex cognitive tasks except for a simple motor tracking task early during the period of sleep deprivation. No significant differences in alertness, heart rate, and root mean square of successive differences in R-R intervals.	Positive or negative depending on task and time

Sakamoto et al. (2009) [15]	Reaction time	Odorless and tasteless gum base	Shortening of reaction time and the peak latencies of event-related potentials (P300 and N100) in second and third session after chewing.	Positive

Smith (2009) [16]	Mood, alertness, intelligence test, and short term and working memory	Volunteer's preferred gum	Improvement of intelligence test. No significant difference in memory. Increased alertness at the end of the test session.	Positive

Scholey et al. (2009) [21]	4 tests including memory, attention, and mood	Volunteer's preferred gum	Overall better performance on the cognitive tasks. Increase of alertness and reduction of stress and salivary cortisol.	Positive

Smith (2009) [18]	5 tests on alertness and mood	Caffeinated gum or placebo gum	More positive mood after chewing and at the end of the study regardless of caffeine. Higher alertness after chewing. No significant difference in alertness without caffeine.	Not significant

Sakamoto et al. (2009) [14]	Reaction time	Odorless and tasteless gum base	Shortened reaction time at third trials after chewing. Increased contingent negative variation (CNV) at second and third trials after chewing. No significant differences in movement-related control potentials.	Positive

Tänzer et al. (2009) [30]	16-minute concentration test	Sugar-free fruit gum	Improvement of concentration performance with time in 8-9-year-olds.	Positive

Smith (2010) [17]	10 tests on memory, attention, and mood	Spearmint or fruit gum	Improvement of alertness and selective and sustained attention. Shortened reaction time; this effect became bigger as the task became more difficult. Increase of heart rate and saliva cortisol.	Positive

Tucha et al. (2010) [42]	Vigilance and sustained attention	Sugar-free spearmint gum	Deterioration of vigilance performance in both healthy children and children with ADHD (mean age 10.8 years). No significant difference in sustained attention.	Negative

Tucha and Simpson (2011) [29]	Sustained attention	Sugar-free spearmint gum	Detriment on sustained attention in earlier stages of 30-minute task and benefit on sustained attention at later stages.	Negative early to positive later within 30-min period

Johnson et al. (2011) [25]	4 tasks including memory and attention, mood, and alertness	Regular chewing gum	Increase of self-rated alertness and stress. No significant differences in task performance and saliva cortisol.	Positive

Onyper et al. (2011) [41]	5 tasks including memory and attention	Spearmint or doublemint gum with or without sugar	Improvements of performance of several tasks in chewing for 5 minutes prior to testing. No significant differences in chewing throughout testing.	Positive in chewing prior to testing

Sketchley-Kaye et al. (2011) [26]	Acute stress task, mood, anxiety, and alertness	Regular chewing gum	Attenuation of state anxiety and increase of alertness under condition of acute social stress task. No significant differences in contentedness or calmness.	Positive

Allen and Smith (2012) [31]	4 tasks on attention and mood	Volunteer's preferred gum	Increased alertness, mood, and performance of attention test and shortened reaction time. Initial extended vigilance reaction times were shortened after trials.	Positive, but some tests showed negative effects initially

Allen and Smith (2012) [38]	4 tasks on attention and mood	Volunteer's preferred gum	Increased reported alertness for positive and neutral demand characteristics. Improved selective attention. Reduced performances of two attention tasks only at specific time of trials. Better response organization on categoric search task when demand characteristics and pretest attitudes to gum were both negative.	Positive, but some tests showed negative effects at specific time of trials

Hirano et al. (2013) [12]	Alerting and executive control	Odorless and tasteless gum base	Shortened reaction time. No significant differences in alerting and conflict effects. Higher activations in the anterior cingulate cortex and left frontal gyrus for the executive network and motor-related regions for both attentional networks	Positive

Johnson et al. (2013) [28]	Sustained attention, alertness, and mood	Cool Breeze gum	Improved attentional task performance. Higher alertness and mood. Shortened response times. No time-on-task effect.	Positive

Morgan et al. (2014) [32]	Short-term memory, vigilance, and mood	Sugar-free spearmint gum	Attenuated time-dependent decrement on both performance and subjective alertness. Shorter correct reaction time in the latter stage of the task.	Positive, especially in the latter stage of the task

Allen et al. (2014) [35]	Vigilance and mood	Gum base	Shortened reaction time and increased rate of hits. Heightened heart rate during chewing. Increased EEG beta power at F7 and T3 immediately after chewing.	Positive
